# Nice Prescriptions

**Published:** 1887-03

**Authors:** 


### NICE PRESCRIPTIONS.

Through the courtesy of Messrs. R. L. Palmer & Co., Druggists, of this city, we have just received from Messrs. John Wyeth & Brother, of Philadelphia, a package containing samples of most elegant preparations. Amongst these, we notice with particular interest Elixir Phosphate Iron, Quinine and Strychnia; Beef, Iron and Wine; Elixir of Gentian, with Tincture of Chloride of Iron; Compressed Lozenges of Pepsin and Charcoal, with Magnesia and Ginger; Compressed Lozenges of Compound Licorice Mixture; Fluid Extract of Ergot; Compressed Tablets of Dover's Powder, Peptonic Pills, Soda Mint, Muriate of Ammonia, Chlorate of Potash, Chlorate of Potash and Borax; Compressed Powders of Saccharated Pepsin; Compressed Powder of Boracic Acid.

This is not the first time we have been favorred with preparations from this excellent house. We have no hesitancy in testifying to the *purity*, *activity* and *beauty* of Wyeth's Preparations. They can be had at the store of Messrs. R. L. Palmer & Co., Druggists, 18 Kimball House, Atlanta.

